# Effects of intrahipocampal nmda on re-extinction of an aversive emotional memory task in rats

**DOI:** 10.1192/j.eurpsy.2023.1278

**Published:** 2023-07-19

**Authors:** E. P. Ruiz Gonzalez, D. M. Gómez Ordoñez, L. F. Cárdenas, M. N. Muñoz Argel

**Affiliations:** 1 Universidad Pontifica Bolivariana, Montería; 2 Universidad de los Andes, Bogotá, Colombia

## Abstract

**Introduction:**

N-Methyl-D-aspartate (NMDA) receptors are involved in learning and memory. It is known that ventral hippocampus is a crucial structure involved in emotional memory formation mainly for fear and anxiety situations. The aim of this research is to identify the effect of the stimulation of ventral hipocampal NMDA receptors on the reextinction of an aversive emotional memory task. NMDA (0.2 ug/μl; 0.2 μl) or saline (0.9 %; 0.2 μl) was bilateral and locally administered in the ventral hippocampus of male Wistar rats, before the re-instatement trial.

The experimental group consisted of 10 animals and the control group by 9 subjects. The results suggest that the activation of ventral hipocampal NMDA receptors induces an increase in the time needed to re-extinguish the conditioned fear, suggesting a possible potentiating effect on re-installation.

**Objectives:**

To evaluate the effect of NMDA at the intrahippocampal level, on the reinstatement and re extinction of a conditioned fear response in male Wistar rats.

**Methods:**

This study is experimental, where two groups of adult male Wistar rats were used. The bilateral cannulas was implanted, the animals were injected intraperitoneally with a mixture of ketamine (Rotexmédica) and xylacin (Bayer; 75 mg/Kg and 5 mg/Kg), respectively, then the animals were placed in a stereotaxic apparatus (Narishige) and injected with veterinary antibiotic.The (21G) caliber cannulas were bilaterally implanted in HPv at the following coordinates: AP = -5.2 mm relative to Bregma; ML = ± 5 mm in relation to the midline and DV = 5.1 mm in relation to the skull and according to the atlas (Paxinos & Watson, 1985).

**Results:**

It was observed that in the first phase of extinction there were no statistically significant differences between the two groups, experimental and control, as in the second phase of extinction. The results obtained for the re-extinction phase 1 and 2 showed that there were significant statistical differences between the groups. This difference was only evident in the first three minutes in the two phases of re-extinction.

**Image 2:**

**Figure:**
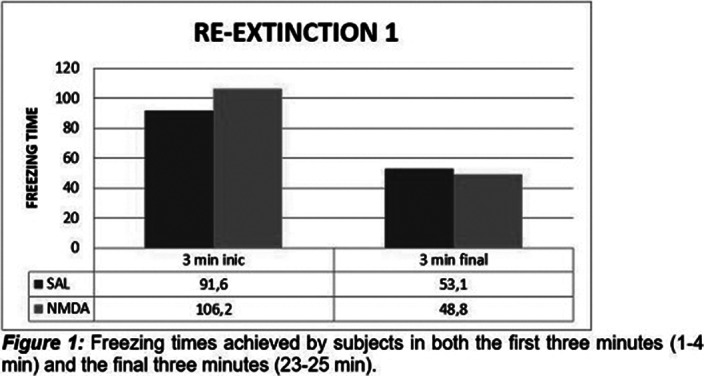

**Image 3:**

**Figure:**
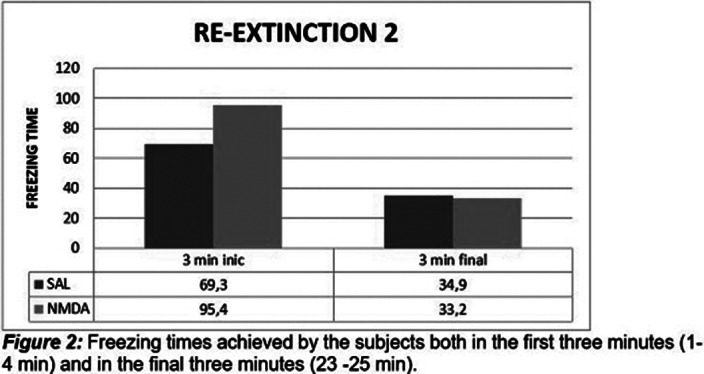

**Conclusions:**

Statistically significant differences were observed between the two groups, in the phases of re-extinction, seeing a longer time of the freezing response in the experimental group, as an effect of the application of NMDA in the ventral hippocampus (HPv), which suggests that this substance has a memory-enhancing effect, and therefore contributes to increasing the permanence of the fear response. It should be noted that this difference was only evident in the first three minutes in the two re-extinction phases. These results may be related to other studies where it has been shown that LTP is dependent on the N-methyl-D-aspartate receptor in the CA1 region *in vivo* (Zhong, Cherry, Bies, Florence, & Gerges, 2009)

**Disclosure of Interest:**

None Declared

